# Intraosseous Cavernous Hemangioma of Inferior Turbinate: A Rare Case Report

**DOI:** 10.1155/2011/431365

**Published:** 2011-09-13

**Authors:** M. N. Akiner, M. T. Akturk, M. Demirtas, E. O. Atmis

**Affiliations:** Department of Otolaryngology-Head & Neck Surgery, School of Medicine, Ankara University, 06100 Ankara, Turkey

## Abstract

*Objectives*. To investigate hemangiomas in the differential diagnosis of the nasal cavity neoplasms, even though it is an extremely rare mesenchymal tumor of the nasal cavity, and the world literature was reviewed. *Case Report*. A 57-year-old woman applied to our department with a 5-year history of left-sided nasal obstruction without history of epistaxis, nasal packaging, or facial trauma. Anterior rhinoscopic examination revealed a mass originating from inferior turbinate that completely obstructs the left nasal cavity. Paranasal computed tomography (CT) showed that the bony mass originated from the anterior part of the left inferior turbinate. Surrounding tissues were normal, and there was not any erosion or destruction. Mass was excised by the endoscopic approach. Histological diagnosis was reported as osseous cavernous hemangioma. *Conclusion*. Hemangiomas are a rare cause of intranasal masses. Its unusual site and masked presentation makes the differential diagnosis difficult. When a bony hard, well-shaped mass was seen in the nasal cavity, the possibility of intraosseous hemangioma must be remembered.

## 1. Introduction

A wide range of tumors can be seen in the nasal cavity. Nonepithelial tumors are uncommon and most of them are benign [1]. Hemangiomas of the nasal cavity compose of less than 20% of all benign nasal cavity tumors [2]. Nasal cavity hemangioma generally arises from the soft tissues of the nasal cavity. Hemangiomas also occur as solitary lesions in bones and account for less than 1% of all primary bone tumors. Intranasal hemangiomas rarely arise from osseous structures. Intraosseous hemangiomas usually occur in the vertebral column and skull bones. Intraosseous cavernous hemangioma of the inferior turbinate is extremely rare; only two cases have been reported in the literature. Herein we present a case of intraosseous cavernous hemangioma of the inferior turbinate in this paper.

## 2. Case Report

**Figure 1:**
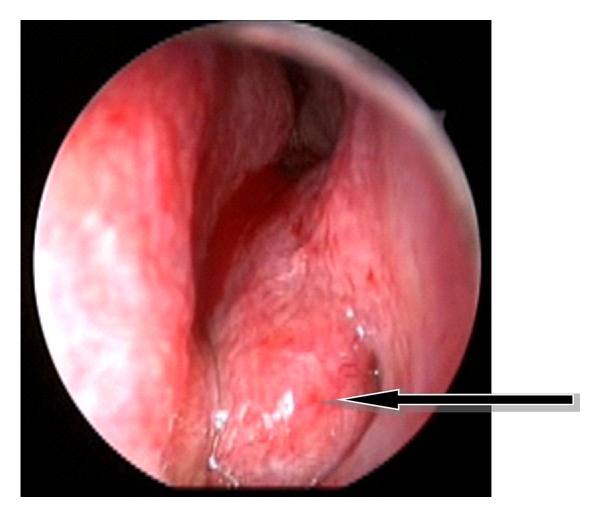
Anterior rhinoscopic view showing the mass arising from the left inferior turbinate.

**Figure 2:**
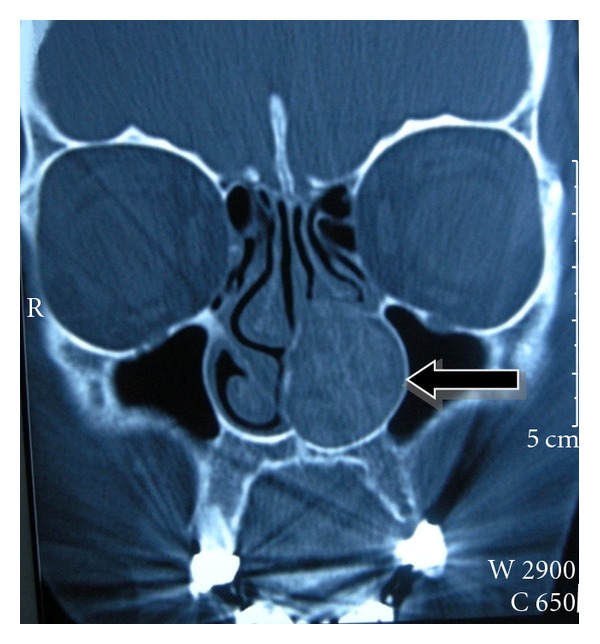
Paranasal sinus computed tomography coronal section showing the bonny mass originated from the inferior turbinate that filled the left nasal cavity.

**Figure 3:**
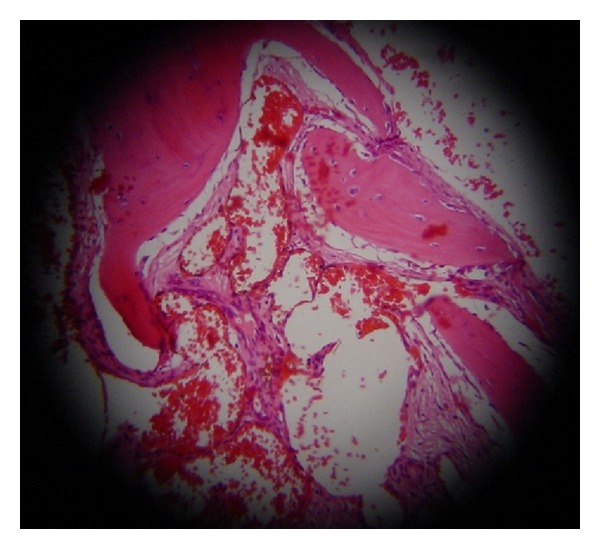
Histological examination: Mass that composed of blood-filled, thin-walled vessels between the bony trabeculae. The lesion was diagnosed as intraosseous cavernous hemangioma. (H&E; ×40).

A 57-years-old woman applied to our department with a 5-year history of left-sided nasal obstruction. She had no history of epistaxis, nasal packaging, or facial trauma. Anterior rhinoscopic examination revealed a mass originating from inferior turbinate that completely obstructs the left nasal cavity. The mass was bony hard and covered with intact, normal colored, and nonhypervascularized mucosa (Figure 1). No other significant findings were seen in the head and neck examination. Paranasal computed tomography (CT) showed that the bony mass originated from the anterior part of the left inferior turbinate. Surrounding tissues were normal and there was not any erosion or destruction. Only minimal deviation of the nasal septum was observed (Figure 2). We did not perform magnetic resonance imaging (MRI) or embolisation because it was considered as fibrous dysplasia at first. Vascular pathology was not supposed. Mass was excised by the endoscopic approach under general anesthesia. The mass involved anterior part of the inferior turbinate. Lesion was 5 × 4 cm in size, hard with bony particles, and covered with intact normal mucosa. There was a significant hemorrhage but it was controlled with nasal packaging. Blood transfusion was not needed. After excision of the lesion, nasal cavity was packed with merocel pad. The packing was removed on the second postoperative day. The postoperative course was uneventful. Microscopically, the tumor was composed of bony trabeculae and anastomosing vascular channels of cavernous pattern. Histological diagnosis was reported as osseous cavernous hemangioma (Figure 3). There was no evidence of recurrence at 1-year followup.

## 3. Discussion

Hemangiomas are benign vascular tumors, and it can occur in any tissue that includes vascular component like skin, mucosa, muscles, glands, and bones. Head and neck region is a common site for hemangiomas but nasal cavity is uncommon localization. The most common sites for nasal hemangiomas are the nasal septum (65%), lateral wall (18%), and vestibule (16%). Hemangioma arising in the turbinate is reported previously; however, most of them arise from the mucosa. Intraosseous hemangiomas constitute less than 1% of all primary bone tumors, and the most common site is vertebral column [2, 3]. Hemangiomas histopathologically have two types as capillary and cavernous. Although cavernous hemangiomas of the nasal cavity are uncommon, most intraosseous hemangiomas show a cavernous pattern. Intraosseous cavernous hemangiomas of the nasal cavity are extremely rare. Only two cases of cavernous hemangioma within inferior turbinate bone have been reported in the English literature [1, 2].

The etiology of intraosseous cavernous hemangioma is unclear. Local trauma and menopause are proposed causes although not proven [2, 4, 5]. In this case, there was no history of facial trauma and nasal packaging but the patient was in menopausal period. Osseous cavernous haemangioma affects females two times more than males. Diagnosis is difficult. It presents as a slow growing hard mass with regular margins. It usually does not appear as a vascular lesion like bluish purple discoloration, spontaneous hemorrhage, pulsation, and so forth.

At radiographic study osseous hemangioma have characteristic soap-bubble-shaped appearance [6]. Angiography can be used for diagnosis and typically shows increased vascularity in the tumor area [2]. Although cavernous hemangiomas of the nasal cavity are uncommon, most intraosseous hemangiomas show a cavernous patern [2, 3]. Because of its appearance and tomography findings, we think that it may be fibrous dysplasia and we did not performe MRI or angiography for hemangioma. 

Treatment options of intraosseous hemangioma include surgery, radiotherapy, sclerotherapy, and embolization. Complete surgical excision is mainstay of treatment and also plays a role in definite diagnosis [5, 6]. Although radiotherapy is a good treatment choice for hemangiomas, long-term side effects, such as malignancy, region growth impairment, and scarring, make it an unfavorable treatment modality. Therefore, radiotherapy is only used for unresectable lesions [2, 5, 6]. Transarterial embolization and sclerotherapy can be used but these are palliative procedures [2].

## 4. Summary

In summary, we report a rare case of intraosseous hemangioma of the inferior turbinate. Its unusual site and masked presentation makes the differential diagnosis difficult. When a bony hard, well-shaped mass was seen in the nasal cavity, the possibility of intraosseous hemangioma must be remembered.
